# Natural Selection, Childrearing, and the Ethics of Marriage (and Divorce): Building a Case for the Neuroenhancement of Human Relationships

**DOI:** 10.1007/s13347-012-0081-8

**Published:** 2012-07-05

**Authors:** Brian D. Earp, Anders Sandberg, Julian Savulescu

**Affiliations:** Oxford Uehiro Centre for Practical Ethics, University of Oxford, Suite 8, Littlegate House, St Ebbes Street, Oxford, OX1 1PT UK

**Keywords:** Neuroenhancement, Love drugs, Evolution, Marital autonomy, Default natural ethics, Marriage, Values, Oxytocin

## Abstract

We argue that the fragility of contemporary marriages—and the corresponding high rates of divorce—can be explained (in large part) by a three-part mismatch: between our relationship values, our evolved psychobiological natures, and our modern social, physical, and technological environment. “Love drugs” could help address this mismatch by boosting our psychobiologies while keeping our values and our environment intact. While individual couples should be *free* to use pharmacological interventions to sustain and improve their romantic connection, we suggest that they may have an *obligation* to do so as well, in certain cases. Specifically, we argue that couples with offspring may have a special responsibility to enhance their relationships for the sake of their children. We outline an evolutionarily informed research program for identifying promising biomedical enhancements of love and commitment.

## Introduction

### Love Drugs and Marital Autonomy

In a previous paper^1^ in 2008—on the science and ethics of “love drugs”—two of us (Julian Savulescu and Anders Sandberg) made a case for the neuroenhancement of love and marriage. We introduced the principle of *marital autonomy*, according to which couples “should be free to shape their relationship in the way which best fits them” (p. 37), including through the use of pharmacological substances.

The argument was liberal. In general, individuals should have the freedom to alter their own brain states—through drugs or other means—in order to pursue their personal goals or realize their conception of the good life, so long as they do not harm or infringe upon the rights of others. Marital autonomy extends this sort of reasoning to the activities of individuals who are in committed relationships and who wish to give their mutual love a “helping hand” through science-based neurochemical intervention.

Why might couples choose to enhance their relationships in this way? We gave several answers. There are cultural and religious reasons: love drugs could promote fidelity in settings where monogamy is considered a virtue, for example. There are health reasons: loving marriages reduce stress, promote longevity, and so on, and love drugs could foster marriages of this (salutary) kind. There are hedonic reasons: love drugs could contribute to an enjoyable and satisfying sex life, pleasurable in its own right, but also important for healthy relationship functioning: Viagra (to pick one relevant example) can surely be counted as a “love drug” and is already on the market. And there could even be intrinsic reasons: some people think that love is *inherently* good or beautiful, so might desire to sustain or enrich it by even unconventional means. In short, we argued that couples should be at *liberty* to use love drugs, and that they may have several good *reasons* to do so as well.

### From Freedom to Duty

In this article, we wish to expand our case for the chemical enhancement of love. We will do so by shifting our attention from reasons based on liberty and marital autonomy (couples should be *free* to use love drugs, if they so desire) to reasons based on duty and marital obligation (some couples may have a *moral responsibility* to use love drugs, at least under certain conditions). Let us make clear right away that having a responsibility to perform some action does not entail that it should be mandatory or legally coerced: we will not suggest that anyone should be forced to take love drugs—or any drugs—against their will.

Instead, we think that some couples, recognizing the marital responsibilities we argue for and seeing how love drugs could help to fulfill them, may *choose* to enhance their relationships pharmacologically for that reason. Others may choose not to or may seek to meet their responsibilities through other means.

But how could someone have a *duty* to take love drugs at all? There is a general argument and a more specific one. In the case of marriages *generally*,^2^ the individuals involved have voluntarily placed themselves under a mutual oath to stick together “for better or worse” and “until death do us part.” The relevant duty is simply to honor that marital oath with the full strength of one's efforts, instead of abandoning it too easily when things go “worse.”

Now, clearly the details of what constitutes “too easily” in any particular case might be very difficult to pin down. We will not offer a general prescription here. Certainly divorce is sometimes appropriate, and may even be morally required (when abuse is involved, for example), and we do not advocate putting a chemical band-aid on a violent or broken marriage: that would be a dangerous mistake. Yet in broadest terms, granting the considerations just laid out, one type of argument for “duty” runs like this:

1. In making a solemn promise to stay faithfully wedded to another human being for the duration of one's life, no matter what future circumstances may unfold, a person becomes obligated to fulfill that promise by *every reasonable effort*.
2. As love drugs become safely and cheaply available; if they could be shown to improve love, commitment, and marital well-being—and thereby lessen the chance (or the need) for divorce; if other interventions had been tried and failed; and if side-effects or other complications could be minimized, then using them might in some cases fall within the bounds of “every reasonable effort.”

### The Special Obligation of Parents

That is the most general line of reasoning we can see for a duty to take love drugs, and there is more we could do to spell it out. But our concern in this article is more specific. For reasons to be given shortly, we will build our case not on the back of binding oaths or marital duties per se—although we think these considerations are important, and that they do factor in—but on the moral obligations entailed by a special subset of romantic relationships, namely those involving offspring. Our focus is on the relationship–obligations of *parents*. We argue:

1. Parents have an obligation to protect their children from harm, all else being equal.
2. Marriage breakdown, and especially outright divorce,^3^ is harmful to children.^4^
3. Therefore, parents have an obligation (all else being equal) to preserve and enhance their relationships—for the sake of their offspring.

Obviously, this is not an argument for love drugs—yet. All it shows is that parents may have a special responsibility to improve their relationship *by some means*, and that method could just as well be marriage counseling as neurochemical enhancement. In order to make a duty-based case for love drugs in particular, then, we would have to show something like the following:

1. Parents have an obligation (all else being equal) to preserve and enhance their relationships for the sake of their offspring.
2. In many cases, *the only way* 5 *to do this* is through pharmacological intervention, in conjunction with other more conventional strategies like couple's therapy.

To understand why love drugs, in certain cases, might turn out to be the only way (or the best way) to save a troubled marriage (and thereby protect children from the harms of divorce), consider an analogy to the case of *chronic depression*, and its treatment through neurochemical means, such as with an antidepressant like citalopram.

### “Treating” Marital Difficulties: an Analogy to Depression

In practice, before prescribing any drugs, doctors and clinical psychologists should encourage a depressed person to address her mental health problems through “traditional” means such as “talk” therapy and by making relevant changes to the broader circumstances of her life.^6^ If a patient is depressed because she hates her job, for example, or is not getting enough exercise, or engages in destructive patterns of thought, her therapist might encourage her to find alternative employment, take up jogging, or practice healthier mental habits.

But sometimes the patient is so depressed that making these sorts of changes by sheer dint of will would be too difficult to manage. Her brain chemistry may be so out of order that she requires a dose of medication to “get over the initial hump” of her depression—to get motivated. Once she is in a more balanced state of mind, she may be able to take the additional steps that are needed to address the “bigger picture” issues that are contributing to her mood disorder.

A similar scenario could apply to a relationship. In some cases, traditional counseling may be insufficient to get a couple “over the initial hump” of their marital difficulties, and love drugs could give them the boost they need. This is because love drugs would target, directly, the psychobiological factors which underlie relationship instability, and could do so across a range of common cases, as we will show. To give an example, consider that the ability to communicate effectively—a prerequisite for resolving conflicts and improving relationship quality (e.g., Bray and Jouriles 1995)—is partially influenced by biochemical processes. In one study, Swiss researchers showed that nasally inhaled oxytocin can reduce stress levels and promote more positive communication between couples engaged in an argument (Ditzen et al. 2009). While more work is needed to assess the effectiveness of this type of intervention in the long term (and outside of a laboratory setting), it gives a good idea of what we mean.

Of course, the turmoil involved in any *individual* marriage could be due to an unknown, overdetermined myriad of causes operating at many levels of description. These causes could range from pathological changes in brain chemistry (such as chronically low levels of oxytocin), up through conflicts in consciously held values, to financial disagreements, and so on. Love drugs are not a panacea: we do not suggest that they could “fix” every relationship problem for every couple, even as our understanding of neurochemistry advances—and some marriages may be so dysfunctional that *any* sort of treatment would be bound to fail. Our aim, rather, is to sketch a more general program for research into the neuroenhancement of love and commitment based on typical, and plausibly correctable, patterns of marriage breakdown grounded in molecular-level considerations. Drug-based interventions inspired by this line of research, once carefully vetted through clinical testing and other controlled methods, could then be tailored to the needs of individual couples on case by case basis.

### The Role of Evolution in Explaining and Addressing Marriage Fragility

This project involves an appeal to evolutionary theory that works in two directions. First, we think that an understanding of human evolution can go a long way in helping us to *explain* the initial problem we seek to address in this paper, namely the fragility of contemporary marriages and the attendant harms (including harms to children) stemming from divorce and relationship breakdown. In simplest terms, natural selection did not organize our mating strategies, nor our psychosexual natures, with modern relationship values in mind—and the resulting tension can cause a great deal of trouble. As Symons (1985) puts it, “in analyzing the psychological underpinning of marriage [we should] keep in mind that *Homo sapiens* is the product of evolution by natural selection; we are designed to promote gene, not individual, survival; reproductive, not marital success” (p. 152).

At the same time, we acknowledge that human beings are not mere puppets of our evolved natures: we can “read, write, play bridge, raise cats or Cain instead of children, and do an infinite number of other things that natural selection did not design us to do” (Symons 1985, p. 153). Cultural forces, then, have a very large role to play in any convincing explanation of human behavior, and we are loathe to be too credulous^7^ about—or too strictly tied to—the specific adaptationist accounts of modern mating habits offered up by evolutionary psychologists. Accordingly, we invoke evolutionary theory for a second reason, going beyond mere explanation: we use it to zero in on particular psychological mechanisms and neurochemical pathways likeliest to be involved in forming and maintaining the human pair bond, giving structure to the love drugs research program we outline. To quote Symons (1985) again: “A Darwinian … view of the mind can suggest new questions and inspire research … [and generate findings] that would never have been discovered” without thinking in evolutionary terms (p. 135). The ultimate test of love drugs, of course, is whether or not they *work*—the calculus of natural selection aside—but we think that evolutionary theory can give us a head start in asking productive research questions, pointing us in the direction of the most plausible pharmacological candidates and giving us a sense of how they might reasonably serve to enhance relationships.

### Summing up and Looking Forward

Here is the plan for the rest of our paper. First, we describe the problem in more detail: widespread marriage breakdown and the bad effects of divorce on children. We argue that these bad effects create a special obligation in parents to sustain and improve their relationships, above and beyond the obligation generated by the oath of a wedding vow; and we think that both of these obligations lend support to a “duty-based” case for taking love drugs. We then suggest that a major source of contemporary marriage fragility is a mismatch between our evolved psychosexual natures and our conscious relationship values, exacerbated by the conditions of the environment in which we live. While this tension could be resolved, in principle, by a change in values and/or environment, we think that there are good reasons to leave those dimensions more or less intact, as we explain. Accordingly, we focus our attention on psychobiological changes we could effect through pharmacological intervention. Using evolutionary theory to identify the brain-level chemical pathways likeliest to serve as good targets for such intervention, we outline a program for research into the neuroenhancement of human relationships.

## The Problem: Marriage Breakdown, Divorce, and Their Mal Effects on Children

Marriage^8^ is a fraught institution. On one hand, it represents a lifelong, monogamous relationship ideal and is a central aim in life for many people (Pew Research Center 2010). Yet on the other hand, more than half of marriages end in divorce^9^ (Williams et al. 2005; US Census Bureau 2012)—and often with traumatic consequences for everyone involved. That includes not only the divorcing individuals themselves, but their children (if they have them), their close friends, and their extended families as well (Nichols 1998; Spaht 1998).

That divorce occurs so widely, despite the best hopes of the couples getting married, is reason enough to consider a range of strategies—even seemingly radical strategies like drug-based treatments—to fill the gap between the ideal and the reality. In this paper, we emphasize the effects of divorce on children (and generate a duty-based argument therefrom) simply because the long-term harms they suffer as a result of their parents' relationship failure are not widely recognized. The idea that “the kids will be fine” if divorce occurs—and that what matters most of all is the subjective satisfaction of the married couple—is very much in vogue (Spaht 1998).

Yet, divorce can be devastating for children, in ways that researchers are only recently beginning to understand. This is true even when both of the parents anticipate a happier time for themselves outside the marriage bond (Spaht 1998), and even when they think, in good faith, that their offspring will be better off as well (Horn 1998). This latter notion—though consistent with conventional wisdom—may turn out to be a myth (Wallerstein et al. 2000). In fact, “in comparison with children of intact first marriages, children of divorce suffer in virtually every measure of a child's well-being, whether educationally, economically, physically, psychologically, or emotionally” (Spaht 1998, p. 1554; see a recent review of the evidence by Amato 2010).

Statistics reveal that more than half of all divorces involve minor children, and 40 % of young people will experience parental divorce before they reach adulthood. In the USA alone, over a million children go through the experience each year (Amato 2000). Its mal effects are *cumulative* over the lifetime of the child (Wallerstein and Lewis 1997), leading to intense relationship insecurity and other developmental problems well into adulthood (Wallerstein et al. 2000), and problems occur regardless of the child's age at the time of divorce^10^ up to and including the age of legal maturity (Amato 1993). Finally, children of divorced parents are themselves more likely to divorce, bringing the cycle of suffering fully around (Amato and Rogers 1997). “For the sake of the children,” concludes Spaht (1998, p. 1547) in her wide-ranging review on this topic, marriages involving offspring are in urgent need of strengthening. We agree and we aim to show how love drugs may help with this goal in a later section.

One objection to this line of evidence is that it is mostly correlational: children of divorced parents show a range of deficits compared to children from intact families, but this does not mean that divorce *caused* the deficits seen in the first group. It is possible that one and the same set of parental relationship problems contributed both to divorce and to the documented decrements in child welfare, introducing a major confound. We simply point out that the moral goal is to protect children from harm (all else being equal) at whatever juncture it is being delivered, regardless of whether actual divorce is on the table.

Yet, there is some evidence that divorce itself can harm children, above and beyond the harms stemming from parental conflict or other relationship difficulties existing in the marriage. In order to demonstrate that divorce is *worse* than a doleful marriage—from the perspective of the child—three contrast points are needed: happy marriage, unhappy marriage, and divorce. A seminal study by Forehand et al. (1997) made just this comparison and revealed that “adolescents from the [unhappy marriages] functioned similarly to those who would remain in [happy] intact families, but better than those in the… divorced group, suggesting that differences can be attributed to parental divorce and its accompanying disruption of family processes” (p. 157). A single study cannot be considered conclusive, but Forehand et al. do raise the important point of mechanism: what sort of “disruption of family processes” could be leading to the alleged harms of divorce?

In the first place, “divorce often leads to a partial or complete collapse in an adult's ability to parent for months and sometimes years after the breakup. Caught up in rebuilding their own lives, mothers and fathers are preoccupied with a thousand and one concerns, which can blind them to the needs of their children.” So report Wallerstein et al. (2000, p. xx) in their landmark 25-year study of the long-term effects of divorce on child welfare. They go on to say, “When children of divorce become adults, they are badly frightened that their relationships will fail, just like the most important relationship in their parents’ lives failed… They are no less eager than their peers who grew up in intact families for passionate love, sexual intimacy, and commitment. But they are haunted by powerful ghosts from their childhoods that tell them that they, like their parents, will not succeed” (p. xiii).

### Ethical Implications of Divorce Harms to Children

What does all of this mean for parents who are considering a divorce—not because of a severe and unsolvable crisis, not because of violence or abuse, or fundamental incompatibility, but because of a long train of minor discords, because their love is graying, or because their passion has waned? What if they are simply unhappy? In their book entitled *A Generation at Risk*, Paul Amato and Alan Booth conclude from their research into the effects of divorce on children that “spending one-third of one's life living in a marriage that is less than satisfactory in order to benefit children—children that parents elected to bring into the world—is not an unreasonable expectation” (Amato and Booth 1997, p. 238).

Perhaps Amato and Booth have it right, although the trade-off between parent welfare and child welfare may not be so clear cut as that. In any particular case, it can be hard to determine whether the suffering experienced by a spouse in an unhappy marriage is worse than the harm experienced by a child due to divorce. But there are some important asymmetries between the two cases, so a simple weighing of harms does not give the full accounting.

The parent *elected* to enter the marriage in the first place, as Amato and Booth point out, in full knowledge that it might grow tough; the child did not *elect* to be born and came into the world totally dependent upon parental care. The parent has already gone through childhood and, presumably, adolescence and has thus survived the most vulnerable stages of human development; the child has not. So, we do agree that the flippant abandonment of a marital commitment, due to one's own private sense of lack of satisfaction and under the misguided impression that “the children will be fine,” would be a serious dereliction of one's responsibilities toward one's offspring. But we also acknowledge that there may be certain cases in which it could be morally justifiable for a suffering parent to choose in favor of her own well-being, even if this would harm the child.

But what parent would choose suffering—for himself or for his child—if it could be avoided? Most parents do not *want* to see their marriages fall apart, and they certainly would not *want* to conjure the “powerful ghosts” of relationship failure which Wallerstein et al. described as “haunting” the children of divorce throughout their lives. So, we expect that many unhappy couples, faced with a choice between (on one hand) total relationship breakdown and serious if not severe collateral suffering in their own children and (on the other hand) remaining trapped in a joyless conjugal bond, might be motivated to explore every possible avenue to bringing love and well-being back into their marriages—for their own sakes *as well as* for that of their children.

Love drugs are one such possible avenue. To understand why they may be a particularly *promising* one, it is necessary to step back and consider the deeper roots of marriage instability, as we undertake to do in the following section.

## Getting to the Root of the Problem: the Tension of Nature–Value–Environment

The gulf between marriage-ideal and marriage-reality—and the ensuing high rates of divorce—can be explained in large part by an uncomfortable tension among three major factors:

1. Deep drives and other facts concerning our biological and psychological make-up as human beings. These were forged into our natures over thousands of generations by the Darwinian fires of evolution.
2. Certain widely held social and ethical ideals relating to those drives and facts as they pertain to love and marriage.
3. Various stable background conditions of contemporary life and the environment in which we live—a very *different* environment from the one in which we evolved under the purview of natural selection.

To make the point directly, our (biological and psychological) natures and our (marriage) values do not perfectly line up within our (modern) context.^11^ This discrepancy then leads to a great deal of relationship friction—which should come as no surprise. In the days before we intervened with birth control, biotechnology, and large-scale manipulation of the environment, natural selection was concerned with human well-being only *instrumentally*. That is, it “cared” about our happiness only insofar as it reinforced the sorts of drives and behaviors that maximized ancestral output of healthy babies (see Dawkins 1976; Harris 2010). Our conscious values, in contrast, aim at our flourishing *directly*. They do so in the context of radically different survival pressures, centuries of self-reflective critical reasoning, cultural accrual, and philosophical progress and a substantially altered physical, social, and technological environment. These factors rightly push such purely gene-driven motives as reproductive success into the back seat. As a result, our psychosexual natures (designed by natural selection on the African Savannah) and our moral values (shaped by concerns about well-being, justice, and so on, in the present day) sometimes sharply disagree. And when they do disagree, it is very often our *values* that buckle under the pressure.

We will offer several examples of this phenomenon in the sections below, but here is one preview case to start. For the sake of illustration, we will focus on *one* marriage value for now—the value of sexual fidelity. Consider:

1. *Contemporary (marriage) value:* Most modern marriage vows entail an explicit promise by the couple involved to be sexually exclusive to each other. Sexual fidelity is widely considered a virtue; adultery is considered a serious moral failure (see Winking et al. 2007), even a sin. Indeed, fully 97 % of respondents to one survey reported that married individuals should *not* have sex outside the relationship (Johnson et al. 2002). And this value seems to find justification on utilitarian grounds, at the very least: committed, stable marriages conduce to happiness and well-being on a variety of standard measures (Wilson and Oswald 2005). Infidelity leads to emotional and physical distress, decreased relationship quality (if it can sustain the blow at all—see below), and a list of other ills (Buss 1995). In fact, the most common cause of spousal homicide is a husband's suspicion that his wife has been unfaithful (Daly and Wilson 1988).
2. *Human (biological and psychological) nature:* Growing evidence suggests that our species did not evolve as completely monogamous. Though our ancestors did form cooperative pair bonds, and while fragile, dependent hominid babies required substantial biparental care for approximately the first 4 years of life (Fisher 2000), it is likely that both males and females throughout human adaptive history had sex outside of their principal reproductive alliances. Evolutionary theory suggests that this behavior would be strongly selected, increasing the inclusive fitness of both sexes—though for different reasons (and to different degrees) in each case. Specifically, due to sex-related asymmetries in the minimum parental investment required for offspring survival (Trivers 1972), comparatively wanton promiscuity would further the genetic interests of ancestral human males; comparatively selective promiscuity would further the genetic interests of ancestral human females; and perfect, full-fledged monogamy would have been maladaptive for both. Insofar as we have inherited the anatomical, biological, and psychological architecture of our forebears, we have a hard-wired desire for a (limited) form of primary partner commitment as well as a powerful, sex-lopsided urge for extra-pair copulation over the life course^12^ (see Symons 1987, 1992; Tooby and Cosmides 1992; Buss 1994; Barash and Lipton 1997).

This tension is made worse by our modern environment:

3. *Modern context:* Sex is now decoupled from mandatory reproduction—thanks to birth control technology—making extra-pair copulation less likely to result in unwanted offspring. Condoms in particular reduce the risk of contracting sexually transmitted infections, dropping traditional (direct) costs of infidelity even further. Ease of long distance travel and the ballooning size of social groups increase opportunities for such (comparatively) low-risk love affairs (Greeley 1994). And these opportunities are multiplied over the protracted length of present-day relationships, since we now out-live our ancestors by decades (Westendorp and Kirkwood 1998).

Given this unharmonious set of factors, it can hardly come as a surprise that *at least* one fifth of husbands and *at least* one tenth of wives commit adultery. These numbers—for American couples—range as high as 72 and 54 % respectively, depending on the survey (see Greeley 1994; Allen et al. 2005).

And what is the consequence? Very often the dissolution of conjugal ties. Betzig (1989) found that marital infidelity was the most commonly cited cause of divorce across 160 societies. South and Lloyd (1995) report that at in at least one third of US divorce cases, one or both partners had been romantically or sexually involved with someone outside the relationship prior to marriage breakdown. And Amato and Rogers (1997) conclude from longitudinal data that extramarital sex is a “particularly powerful” causal predictor of ultimate marriage failure (p. 622). Adultery and divorce^13^ go hand in hand, then; and adultery is a foreseeable outcome of the three-part mismatch described above.

So what can be done? We have given a framework according to which three major forces sit in tension: our evolved psychobiologic natures, our considered (marriage) values, and our modern environment; and we have shown one way in which that tension can lead to painful divorce and collateral harm to children. To close the gap between marriage-ideal and marriage-reality, then, we must exert some sort of change along one or more of those dimensions. Let us consider the options.

## What Should We Change: Our Values, Our Environment, or Our Natures?

### Changing Our Values

First, we *could* change our values regarding love and marriage. In general, if our values conflict with our natures, we should go out of our way to confirm that the values are well-justified, and if they are not, we should adopt a set of values that are more harmonious with our evolved psychobiologies. We will explain why in a moment.

To change our values about adultery, we could try to convince people that extramarital sex is “natural” (in the sense that it was selected for in our ancestral environment) and that it should therefore be seen and felt as a non-offense—in effect asking people to commit a form of the naturalistic fallacy and adapt their emotions accordingly. This might reduce divorce rates if affairs no longer provoked so much heartbreak and suffering.

That such a campaign could be effective, of course, is doubtful.^14^ But norms about adultery do differ between cultures and across epochs of time. In seventeenth century England, for example, wives were generally expected to ignore their husbands' “extramarital adventures” since standards for mutual sexual fidelity were practically nonexistent outside of a handful of religious reform groups. Not that the wives were happy with this double-standard or with their husbands' “adventures” in the first place (Coontz 2005).

But a powerful suite of external forces—such as tradition, political alliances, and economic necessity—kept those seventeenth century marriages intact, in spite of any suffering involved. We lack this suite of forces today, so marriages are much more likely to rise and fall on the back of the emotional bonds holding them together (Coontz 2005). This is one major reason why love may need a “helping hand.” And yet, the damaging effects of cheating on the love bond provoke a human universal: jealousy. This adultery-detesting suite of responses may have evolved in both sexes to protect against cuckoldry (in the case of males) and diversion of male resources away from childcare (in the case of females) (Buss 1994), and the women of earlier eras were no exception to this hot-blooded rule. Sexist norms do not nullify human instincts or the pain caused by a philandering spouse.

Romantic jealousy, then, is arguably as much a part of our nature as the impulse to cheat is. Yet, from the perspective of *child* welfare—which is central to the duty-based case we are trying to make—jealousy^15^ is much easier to harmonize with other values. This is because it keeps the parents' attention focused on each other, and on their childrearing obligations, and raises the cost of giving in to sexual temptation. Indeed, extramarital sex often leads to extramarital love (Buss 1995), and hence, the formation of a strong bond that could take time and energy directly away from existing offspring.

The seventeenth century is a poor guide (in any case) to male–female relationship ethics in the present day. Even if patriarchal conventions that privilege (male) promiscuity may resonate better with basic facts about human biology, they are certainly not in tune with modern ideals about mutual respect between individuals, gender equality, and so on. Natural does *not* (automatically) entail good, a point which cannot be made too often when evolutionary psychology and ethics are being discussed in the same paper.

Of course natural does not (automatically) entail *bad*, either. It does make sense, *ceteris paribus*, to argue for values and norms which are consistent with our evolved psychobiologies, as we stated at the top of this section. We call this idea the principle of *default natural ethics*. In the following subsection, we will introduce and justify this principle and then return to our ongoing illustration involving adultery to spell out just what it means for marriage values in the present day.

### When Natural Really Is Good: the Principle of Default Natural Ethics

Why should we adopt norms—all else being equal—that are consistent with our evolved natures? The reason is that it helps us to escape the various harmful effects of misguided prohibitions and repression—whether enforced through law or custom—on individual and societal well-being. Erich Fromm captured this idea eloquently (if sexistly with respect to pronouns) in his 1947 treatise, *Man for Himself: An Inquiry into the Psychology of Ethics*:

If man were infinitely malleable then… norms and institutions unfavorable to human welfare would have a chance to mold man forever into their patterns without the possibility that intrinsic forces in man's nature would be mobilized and tend to change these patterns. Man would be only the puppet of social arrangements and not—as he has proved to be in history—an agent whose intrinsic properties react strenuously against the powerful pressure of unfavorable social and cultural patterns. In fact if man were nothing but the reflex of culture patterns no social order could be criticized or judged from the standpoint of man's welfare since there would be no concept of ‘man’ (pp. 31–31).

Fromm is right as far as he goes, but what are the implications for practical ethics? Evolutionary psychologist Marc Hauser (Hauser 2006)—writing about the “intrinsic property” of our evolved *moral* instincts in particular—spells it out in this way:

It is clear that in the arena of medicine [as well as law and] in so many other areas where moral conflicts arise, the policy wonks and politicians should listen more closely to our intuitions and write policy that effectively takes into account the [natural instincts] of our species. Taking into account our [instincts] does not mean blind acceptance. It is not only possible but likely that some of the intuitions we have evolved are no longer applicable to current societal problems. But in developing policies that dictate what people ought to do, we are more likely to construct long-lasting and effective policies if we take into account the intuitive biases that guide our initial responses to the imposition of social norms (p. xviii).

Putting Fromm and Hauser together, then, we arrive at the following idea. As we have argued elsewhere (see Savulescu and Earp 2011; Earp et al. 2011): human nature should be managed only very carefully, toward well-justified moral goals, based on a keen understanding of our evolved psychobiologies, and always with an empirical eye trained on the real world outcomes of any intervention. Otherwise Fromm's warning that our natures will “react strenuously” against the external impositions—with potentially disastrous side effects—may come swiftly into play.

Sexual repression is a case in point (see Ryan 2010; Ryan and Jetha 2010). It has been argued that Puritanical sexual attitudes in the USA, coupled with abstinence-only sex education policies, lead to such “backfiring” ills as *higher* teen pregnancy rates and *increased* transmission of STIs among American religious youths (Stanger-Hall and Hall 2011). More sex-positive cultures—cultures, that is, that do not regard sex among young, unmarried persons as inherently immoral—exhibit much lower rates of both, as well as greater psychosexual health: Holland is the paradigm example (de Waal 2010). Some researchers have gone so far as to draw a straight line from sex repression and denial of bodily pleasure (as taken to the extreme in some Middle Eastern cultures) to violence, including sexual violence, and even apparently unrelated acts of terrorism (Prescott 1975). And the recent scandal of child sex abuse in the Catholic Church has been linked directly to norms of celibacy in the priesthood (Berry 1992; Plante 1996). Suppressed natural drives creep out. Sometimes the consequences are dire.

It is only when biology-consistent norms about human behavior lead to third party harms or other violations of firmly grounded moral standards (such as justice or equity) that laissez faire policies should be reconsidered and the costs and benefits of any regulatory side effects duly calculated and weighed. Rape, for instance, is certainly natural—it exists in our primate relatives and may have been adaptive for low-status ancestral human males (Wrangham and Peterson 1997); but we should in no sense be “hands off” about rape. We should condemn it wherever it occurs and try to prevent it whenever possible.

What does all of this tell us about adultery? We know that adultery causes harm—in the form of severe emotional trauma, relationship breakdown, and oftentimes divorce; and we know that divorce itself is harmful, on balance, to children when they are involved. We know, too, that the emotional harm associated with adultery is built in through jealousy, and we have evidence that the “green-eyed monster” was designed to keep parental resources focused on existing offspring. Jealousy, then, as opposed to adultery, is consistent with values aimed at keeping families intact. In addition, a hands off adultery norm might also raise concerns about equity, since men are much more likely than women to desire extramarital sex in the first place (Buss 1994). Finally, convincing 97 % of the population to reverse a cornerstone social value seems unlikely, to say the least. For reasons both practical and principled, then, the norm against adultery is probably worth retaining on a broad scale—however “unnatural” absolute sexual fidelity may in fact be. On the principle of default natural ethics, the “all else equal” clause does not hold up.

That leaves just two dimensions on which to maneuver in order to close the marriage-ideal–marriage-reality gap and protect vulnerable children from divorce. (Remember that we are focusing on a *single* value—fidelity—as an example; other marriage values may be more amenable to productive reconsideration, as we will discuss later on). Let us now consider possible changes on the context dimension—that is, possible changes to facts about our environment and modern life.

### Changing Our Environment

What could we change about our modern environment to make adultery and divorce less prevalent? We would not want to backpedal on such new-found goods as improved health and nutrition, which lead to greater longevity—even though a longer life means more opportunities, over time, for extramarital sexual temptation.^16^ And we would not want to advocate social isolation for married couples, reduced travel, or moving back to small village communities so that the spying eye of neighbors could root out bad behavior. The trade-offs in each of these cases would be very hard to justify. So, we shall have to consider altering variables on some other aspect of modernity, taking things like good health, airplanes, and living in cities for granted. If changes in our material domain would be mostly regressive, and if we are holding our values constant for the time being, let us turn to the socio-legal domain.

We could pass laws making divorces much harder to obtain, as has been tried in the US state of Louisiana (Nichols 1998). Or we could make adultery itself illegal, as it is in Pakistan, for instance, where it is punishable by death under the 1979 Hudood Ordinance (Kahn 2006). We could (in theory) reduce access to birth control, so that extra-pair sex involved greater risks of undesired side effects (but that would conflict with other values we have about female reproductive choice^17^). We could impose heavy fines on anyone who had sex with someone other than his or her spouse, as a deterrent; and so on. These measures would ideally reduce divorce rates while *preserving* anti-adultery norms by decreasing the extramarital affairs themselves.

If they did not simply backfire, that is, or go largely ignored. Yet heavy-handed regulation of human mating arrangements typically has precisely these effects (Davis 1985; Coontz 2005). As we have already pointed out, concurring with Eric Fromm, people frequently behave in ways that are consistent with their deepest drives and impulses, moral ideals notwithstanding, and efforts of the state be damned.

We should not be too quick, of course, to dismiss legislative or social policy changes aimed at promoting fidelity—especially when children are involved. And if legal maneuvers cannot eliminate divorce entirely (or ethically), a reduction in rates, on balance, would still be a worthy result. But there is always a tradeoff. To quote Fromm again, “Man can adapt himself even to [unnatural] conditions” although “in this process of adaptation he develops definite mental and emotional reactions which follow from the specific properties of his own nature” (Fromm 1949, p. 32). He goes on to give a relevant example:

Man can [even] adapt himself to cultural conditions which demand the repression of sexual strivings [such as norms about fidelity]… He can adapt himself to almost any culture pattern, but in so far as these are contradictory to his [evolved] nature he develops mental and emotional disturbances which force him eventually to change these conditions since he cannot change his nature (Ibid.).

But can he really not? What if he could change his nature? This is the starting point in our case for neuroenhancement. Fromm was writing when the scientific study of human psychology was in its very infancy, when our knowledge of brain chemistry was essentially nil, and when attempts to modify the cerebrum were limited to electroshock therapy and Frankenstein-like lobotomies (White 2011). For Fromm to take human nature as a constant, then, was simply good practical philosophy. But Fromm was a product of his time, and we have come a long way in the last half century. Recent work in human enhancement suggests that we are (or will soon be) able to intervene directly in the psychobiological systems underpinning such diverse and complex phenomena as moral decision-making (Persson and Savulescu 2008), mathematical ability (Cohen Kadosh et al. 2010), learning and memory (Joshi and Parle 2006), and—most relevant to the present argument, of course—lust, attraction, and attachment (Savulescu and Sandberg 2008).

### Changing Our Natures

So what if we could *keep* our modern values, and *keep* our modern environment, and bring our very natures into line with both? Could we eliminate adultery using knowledge of human neurophysiology? We certainly have the capacity to alter hormone levels controlling the human sex drive. What if we could supplement marriage counseling sessions with prescription love drugs—chemical interventions designed to improve commitment and bonding? This sort of radical approach, like the others we have just entertained, has advantages and disadvantages. Of course, there are better and worse changes that could be made along any of the above dimensions and more and less plausible ways to effect them. Changes could be made in concert across all three. In this article, however, we are focusing on just this third dimension—on how to intervene at the level of the brain for the sake of improving human relationships.

We choose this focus for two reasons. First, it is a method for bolstering marital well-being that has received almost no attention in the published literature (but see Savulescu and Sandberg 2008), and yet the divorce crisis, and its detrimental effect on children, is critical enough to justify outside-the-box thinking for how to address it. But our argument is not *merely* outside-the-box. Indeed, we think that neuroenhancement may be particularly effective in improving modern relationships compared to (or in addition to) more traditional methods such as wide-scale legislation or social policy reforms or narrow-scale methods like couple's therapy and relationship “self-help” manuals. This is because love drugs, properly administered and tailored to the specific needs of individual couples, could address the psychobiological *root* of so much marital discord. It could do so without requiring massive social changes, blunt catch-all legislation, or dubious reconsideration of basic marriage values such as fidelity and gender equality. And while neuroenhancement would not replace marriage counseling and other self-help methods, it could certainly supplement and improve those well-worn measures to good effect.

But before jumping to chemical solutions to the problem of divorce, we should consider the other marriage values (besides fidelity) that are in conflict with our natures. That is the task of the following section.

## There Is More to Divorce than Adultery: the Problem of Love-Based Marriages

We began this essay with a cursory gesture at “the marriage ideal” and we mentioned just two of its features: that marriages should be lifelong, and that they should be monogamous. We have invested a considerable amount of time looking into this second aspect—the norm of sexual fidelity—but there is obviously much more to the crisis of divorce than sex outside of marriage. What else is in the picture? Various sources suggest that we are dealing with a moving target, as Western marriage values are in a state of relative flux (e.g., Coontz 2004), and certainly there is no universal agreement on the topic at any time; but some current, general features can be given nevertheless.

According to the basic stereotype, contemporary Western marriages should have the following hallmarks. They should be entered into freely by (two) autonomous, consenting individuals; they should be initiated and sustained by the power of romantic love, and they should conduce first and foremost to the personal happiness of the married individuals themselves (Coontz 2004; Spaht 1998). While offspring are often desired, marriages are not “for” producing children; hence, it is chiefly a couple's affection and companionship that should bind them together (Lombardo and Lombardo 2008). And as we have already seen, this love-bound fidelity is expected to last, under conditions of perfect monogamy, so long as they both shall live.

Are all of those values worth defending? Do they pass the *default natural ethics* test we introduced above? We have made a case for fidelity, especially when children are involved. And we think that equality, autonomy, and mutual consent are too well-established, in the pantheon of post-Enlightenment ethical principles, to be seriously reconsidered now. What about the idea that marriages should be based on love and the pursuit of happiness? This question deserves a thoughtful answer.

As George Bernard Shaw has written, marriage is an institution that brings together two people “under the influence of the most violent, most insane, most delusive, and most transient of passions. They are required to swear that they will remain in that excited, abnormal, and exhausting condition continuously until death do them part” (Shaw 1908, p. 19). Shaw is obviously embellishing the point, but his central message is clear: it is the fact that we base our marriages on *love*—that “most violent” and unstable of emotions—that is the wellspring of so much trouble. Coontz (2005) has written on this theme somewhat more soberly:

For most of history it was inconceivable that people would choose their mates on the basis of something as fragile and irrational as love and then focus all their sexual, intimate, and altruistic desires on the resulting marriage… Only rarely in human history has love been seen as the main reason for getting married. When someone did advocate such a strange belief… it was considered a serious threat to social order (p. 15).

Marriage has endured for thousands of years, but the centerpiece position for love is indeed a recent development. As late as 1967, two thirds of American college women said they would at least “consider marrying a man they did not love if he met other criteria, such as offering respectability and financial security” (Coontz 2010, p. 1). But even that much concession to extra-love factors, without love itself also being present would seem unusual today. In a recent survey, fully 93 % of married American respondents cited love as a main reason for getting married,^18^ while financial stability was the *least* offered reason, at 31 % (Pew Research Center 2010). Contemporary marriage is founded on the personal desires, goals, and interests of autonomous partners, with their love for one another playing the uncontested linchpin.

Prior to the eighteenth century, things looked very different. For eons till then, marriage had been, at its core, a rather loveless instrument, serving as a sorting hat for the economic and political hierarchy of society. To that end, tying the knot was a way of “raising capital, constructing political alliances, organizing the division of labor by age and gender, and deciding what claim, if any, children had on their parents” (Coontz 2004, p. 977). For most people, marriage was not so much about securing a soul mate, but the right kind of in-laws; and as historian Margaret Hunt has shown, it was not just *a* way, but indeed the main way of transferring “property, occupational status, personal contacts, money, tools, livestock and women” across generations and family groups for centuries (Hunt 1996, p. 151). Love was a non-reason for getting married, and when divorce occurred, “it was more often to get a better set of [family connections] or because of childlessness rather than because love had fled the home” (Coontz 2004, p. 977).

But that was then. We cannot turn back the clock—nor should we want to. The central role of love in marriage is directly tied up in other values we hold so dear in the modern era. As Judith Wallerstein and Sandra Blakeslee have written (Wallerstein and Blakeslee^1995^):

In today's world it's easy to become overwhelmed by problems that seem to have no solution. But we *can* shape our lives at home… The home is the one place where we have the potential to create a world that is to our own liking; it is the last place where we should feel despair. As never before in history, men and women today are free to design the kind of marriage they want, with their own rules and expectations… In our fast-paced world men and women need each other more, not less. We want and need erotic love, sympathetic love, passionate love, tender, nurturing love all of our adult lives. We desire friendship, compassion, encouragement, a sense of being understood and appreciated, not only for what we do but for what we try to do and fail at. We want a relationship in which we can test our half-baked ideas without shame or pretense and give voice to our deepest fears. We want a partner who sees us as unique and irreplaceable… A good marriage can offset the loneliness of life in crowded cities and provide a refuge from the hammering pressures of the competitive workplace. It can counter the anomie of an increasingly impersonal world, where so many people interact with machines rather than fellow workers (p. 5).

Love, then, should be at the heart of any good marriage—we could hardly argue otherwise. But love is fragile. The psychological and biological processes undergirding love were designed by evolution for much humbler ends than keeping a modern marriage running—much less over decades and in the face of the turbulence, unpredictability, and stress of contemporary life. Neuroenhancement can help love rise to the challenge, as we explain in the following sections.^19^

## The Case for Love Drugs: Neuroenhancement as an Adaptive Workaround

Why is neuroenhancement a particularly promising idea for improving love and marriage—above and beyond traditional methods—across a range of cases? Let us return to our analogy involving chronic depression from the beginning of the paper. Sometimes a great deal of “higher-order” suffering (whether for an individual considered on her own, or within the context of a relationship) can be traced to problems in neurophysiology. And while it is of course possible to enhance our brain chemistry through a number of interventions that do not involve pharmacology, one advantage to love drugs is that their effect is direct: it would really be as simple as “swallowing a pill.” Just as with the treatment of depression, then, in some cases that first boost of chemical motivation could make all the difference, considered within a broader therapeutic program.^20^

But “swallowing a pill” can be very dangerous as well. The wrong prescription or an inappropriate dose could spell disaster. The brain systems involved in human love are complex and interlocking, in many ways we do not yet fully understand (Fisher 2000). So, we must be clear about what we are advocating: not the immediate, wanton adoption of pharmacological strategies for improving human relationships, but rather the painstaking use of high-quality research to explore especially promising interventions—based on a keen understanding of the evolutionary roots of marriage instability.

Nick Bostrom and one of us (Anders Sandberg) have given an “evolutionary” heuristic for considering the risks and opportunities involved in enhancing human psychobiology through biomedical means. This heuristic asks why evolution has not already equipped us with the feature we desire—such as the widespread ability to sustain loving and committed relationships over decades in a modern context (Sandberg and Bostrom 2007). If nature did not install the trait because (a) engineering constraints inherent in biology and natural selection would have made it impossible, (b) the trait conflicts with trade-offs that made sense in our ancestral environment but which are no longer relevant, or (c) the trait does not optimize inclusive fitness but rather what we value, then we have a reason to think that the proposed enhancement might not upset some hitherto unknown but important psychobiological function.

In the case of extending the length and strength of human pair bonds, it is clear that biology is *capable* of achieving long-lasting love: we have numerous real-life examples. But we have many counter-examples as well: the brain systems supporting love and attachment evolved to promote cooperative childrearing behaviors over a *limited* stretch of time. Natural selection lacks foresight. It obviously could not anticipate our romantic and parenting needs in the modern era, nor could it “plan ahead” for any of the shifts in adaptive trade-offs occurring over evolutionary time. In consequence, we have a layered attachment system in which older adaptations had to be accommodated by newer ones. Eastwick (2009) explains this set-up in terms of an *adaptive workaround*: new adaptations are designed in part to manage the outcomes of prior selection pressures.

In the sequence of adaptations, the human adult pair bond is a fairly recent arrival on the scene: it is perhaps 0.5–2 million years old (Eastwick 2009). It is younger, then, than sexual desire, mother–child bonding, and a host of other functions related to mate selection and offspring survival. It may have developed in response to the heightened importance of paternal investment in offspring with increasingly large and more complex cerebrums. These burgeoning brains engendered ever slower maturation processes: if parents fell in love and remained together at least during the vulnerable infant period, their fitness would be enhanced (Fisher 1992).

Eastwick (2009) argues that our evolutionary past may have produced a mixed strategy of short-term versus long-term bonding, and systems for both approaches are wired into our brains. Yet, we have only limited control over which system is activated: activation is influenced by unconscious factors such as shifting hormone levels. We might be running on the short-term system when we (or our partners) would prefer a long-term bond—or vice versa. And even if the long-term system is in play, it is only “long term” by ancestral standards, not contemporary ones.

This example shows that the neuroenhancement of relationship stability may be considered an adaptive workaround, designed to deal with evolved systems whose normal operation conflicts with our goals and values in various ways. But instead of being brought about by natural selection, biomedical workarounds are accomplished through human agency and technology. And love drugs in particular meet the evolutionary heuristic outlined above: the situational trade-offs that shaped our pair-bonding brain systems in the environment of evolutionary adaptation have changed radically in the modern era. Deeply rooted, long-lasting love, and healthy, intact families have value to human beings far beyond their usefulness in promoting inclusive fitness.

## How Would It Work? Toward an Evolutionarily Informed Research Program

The core challenge is this: how to make loving pair bonds last better—long enough at least to raise children in a modern environment (see footnote 19). This could be achieved by influencing those bonds directly, as well as by reducing factors that tend to wear them down, and increasing factors that tend to maintain them.

Pair bonds have a clear biological foundation. Underlying human love is a set of basic brain systems for lust, attraction, and attachment that have evolved among mammals. The lust system promotes mating with a range of promising partners; the attraction system guides us to choose and prefer a particular partner; and the attachment system fosters long-term bonding, encouraging couples to cooperate and stay together until their parental duties have been discharged (Fisher 1998; Fisher et al. 2002). These universal systems form a foundation on which the cultural and individual variants of love are built (Gottschall and Nordlund 2006; Jankowiak and Fischer 1992).

Many of the brain regions associated with romantic love in humans are rich in receptors for oxytocin and vasopressin. These hormones buttress many areas of interpersonal interaction: managing social stress, fostering emotion recognition and memory for social information, and so on (Meyer-Lindenberg et al. 2011; Insel et al. 1994; Heinrichs et al. 2009). Their role in the formation of mammalian pair bonds in particular is best understood in the case of voles. Two closely related species employ either a monogamous or a polygamous mating strategy, and the difference appears to depend heavily upon the expression of these hormones. Infusion of oxytocin into the brains of female prairie voles and vasopressin in males, for instance, facilitated pair-bonding even in the absence of actual mating (Cho et al. 1999; Insel and Hulihan 1995; Williams et al. 1994; Winslow et al. 1993).

It has not yet been shown conclusively that human attachment relies on the same hormonal machinery, but it does seem plausible that such a system would be highly conserved (Fisher et al. 2006). Oxytocin is released in other animals by stroking and in humans by frequent partner hugging, which also reduces stress (Light et al. 2005). Administering oxytocin enhances recognition of face identities, but not nonsocial stimuli (Rimmele et al. 2009), and it increases the subjective experience of attachment security in males with insecure attachment patterns (Buchheim et al. 2009). Oxytocin is also involved in nursing behavior, trust, and “mind-reading” (Domes et al. 2007; Kosfeld et al. 2005) as well as in counteracting fear (Kirsch et al. 2005) and increasing readiness for social contact (Heinrichs et al. 2009).

Synthetically boosting oxytocin levels in a marriage context, then, could reduce stress, promote trust, and encourage pro-social behaviors, thereby breaking the negative feedback loops plaguing certain relationships. But it may not be so straightforward. One recent study found that women whose oxytocin levels spiked after a *hurtful* relationship experience had greater anxiety and were less forgiving of the slight compared to women who had lower levels of the hormone (Tabak et al. 2011). So, the context in which any hormone is administered is crucial, and much more research is needed to determine the optimal settings and conditions. As our knowledge concerning the human pair-bond grows more detailed, however, it is likely that specific targets could be found, perhaps influencing the biological representation of a particular attachment object and strengthening it.

Oxytocin and vasopressin are not the whole story. Dopamine, along with oxytocin, is elicited during the early romantic phase of a relationship and during sexual interaction. It acts as learning signal, helping to imprint details of the partner and to associate those details with positive emotions and relationship-promoting habits (Aragona et al. 2006; Liu and Wang 2003; Curtis et al. 2006). This hormone-driven process might be possible to trigger artificially, as has been demonstrated in voles (Williams et al. 1994). If so, such an intervention could be used to reinforce pair bonds when the partners were in close contact—in a marriage counseling session, for example. Other substances that might strengthen pair-bonding under the right conditions include entactogen drugs that promote sociability and an experience of connection with other people (Greer and Tolbert 1986) as well as emotional openness and the reduction of anxiety (Vollenweider et al. 1998). “Ecstasy” or MDMA has been used therapeutically in the development of emotional communication skills (Greer and Tolbert 1990), and it is not implausible that it, or similar drugs, could promote relationship health if administered appropriately.^21^

Just like fidelity, adultery appears to be heavily influenced by brain and even gene-level factors. Variations in a dopamine receptor gene have been found to correlate in humans with infidelity and sexual promiscuity (Garcia et al. 2010). This outcome might be carried out through effects on libido, sensation-seeking, or impulsivity. Similar findings have been reported in rodents (Curtis et al. 2006). Infidelity may also occur through less direct routes—stemming from asymmetrical sexual interests between partners, for instance. As relationships outlast their evolved scaffolding, disparities in sexual desire between men and women tend to expand (Klusmann 2002, 2006): in the typical pattern, men whose libido remains constant while their wives' begins to wane disproportionately seek sexual fulfillment outside the relationship (Buss 1994). By heightening sexual desire in the less aroused partner (by using testosterone, for instance, see Braunstein et al. 2005; Sherwin 2002; Sherwin and Gelfand 1987; Sherwin et al. 1985) or *reducing* it in the more aroused partner, the discrepancy could be minimized, possibly softening a major source of relationship strain. In fact, testosterone levels fall *naturally* in men upon marriage or the birth of a child and *rise* naturally at a relationship's end (to encourage novel mate-seeking behaviors, see Eastwick 2009): deliberately moderating these levels in the right way could promote male parenting and discourage a wandering eye.

In this section, we have given only a sketch of the sorts of hormonal and pharmacological avenues couples may one day pursue to sustain and enhance their relationships: this sort of research is in its earliest stages and much more work is needed. But we think it is time to move beyond merely *describing* the brain systems involved in love, attachment, and commitment: we should begin to think about *intervening* in those systems directly, to give love a helping hand.

## Conclusion

Marriage is a protean institution, having adapted to fit the mores, politics, and economic molding of each generation across cultures and time since the dawn of recorded history. While our marriage values may conflict with our naturally selected drives in the present day, insofar as marriage and marriage-like institutions codify our deepest needs for pair-bonding and meaningful attachment (more important than ever before in the modern world) they can—if properly designed and managed—strike a certain unique *harmony* with our evolved natures. And indeed a successful marriage is one of the greatest predictors of robust well-being that we know of, boosting physical and emotional health, self-reported happiness, and longevity just to start (Wilson and Oswald 2005). Successful marriages are also in the best interests of families, and their breakdown, when children are involved, should be avoided whenever possible. Accordingly, we have argued that much of the fragility in modern relationships could (and should) be overcome by a program of neuroenhancement of love and commitment, based on a keen understanding of the latter's evolutionary roots.

## Footnotes

1 “Neuroenhancement of love and marriage: the chemicals between us” *Neuroethics*, 1:31–44. doi 10.1007/s12152-007-9002-4.

2 Assuming here that they are entered into freely, under conditions of mutual consent, by mature adults. That excludes some forms of arranged marriages, marriages entered into under duress or conditions of coercion, and marriages involving immature individuals or individuals incapable of giving meaningful consent.

3 As increasing numbers of couples have children outside of a formal marriage bond, we should point out that our arguments regarding the obligations of parents apply to these cases as well. The relevant concept, then, is “breakup” rather than “divorce.”

4
*Generally speaking*, as we will show. In some cases, divorce may be better for children than their remaining in a “broken home.” If the parents are so much in conflict as to be outright violent, or if they are unable to keep their fighting and trauma out of view (and earshot) of their family, if conventional treatments have been tried, and none could repair the rift, then “protecting children from harm” might actually *justify* divorce, and love drugs would serve no good end. We restrict our argument to those cases in which divorce is avoidable, would ensue for reasons other than violence or fundamental incompatibility (such as a loss of love or commitment, or milder discord), and whose prevention would save children from the harms of marriage failure. Evidence shows that such cases are more common than many think, as we explain later on.

5 That would be the strongest form of the argument, and we think at least some relationships could meet this standard. But even “the best way” or “a particularly effective way” or “a good way” would justify the use of love drugs in a broad range of cases.

6 This is because neurochemical interventions carry a risk for any number of unwanted side effects (aggression and suicidal thoughts in the case of SSRIs) which could be avoided if the patient's depression were treated by other means. Drugs may also disincentivize a patient from dealing with the “real issues” contributing to her depression by numbing her feelings: she may come to rely on the drug indefinitely to avoid pain without ever tackling the root causes of the problem (dependency). There may be other concerns as well. Nevertheless, sometimes pharmacological treatment for depression is needed when other methods fail; and even if side effects do occur, the patient may decide that they are “worth it” if well-being is improved on balance or else stop taking the medicine if not.

7 We thank an anonymous reviewer for pushing us to clarify the role of evolutionary theory in our argument and for suggesting that we highlight the contentious nature of some of the specific research generated by evolutionary psychologists.

8 In this article, unless otherwise specified, by “marriage” we mean the modern institution of heterosexual marriage as it is conceived of and practiced in Western Europe and North America. We choose this Western-centric, heterosexual focus for a few reasons: it is the model of marriage for which the most data are available in English; it is the most familiar to us anecdotally, and it should help keep the argument focused. Where marriage norms differ from those assumed here, insofar as they exhibit a “mismatch” between the ideal and the reality, and insofar as that mismatch can be (at least partially) explained by appealing to an evolved human nature, then the sort of reasoning we present in this paper will extend outward to those cases.

9 Divorce rates are notoriously hard to pin down (see Amato 2010). The commonly cited 50 % figure derives from the number of divorces per 1,000 people in a given population (in many reports, the US citizenship, based on census figures) compared against the number of weddings per 1,000 people in the same population over the course of a year—a ratio which, in recent decades, has remained at or near 1:2 across a range of samples. Other ways at arriving at the 50 % statistic depend upon *projections* based upon data collected in the 1970s, 1980s, and 1990s, assuming that trends would continue into the present day. Some authorities suggest that divorce rates may be declining in recent years; other authorities suggest that they are rising. Taking all of these considerations together, however, divorce expert Paul Amato has concluded that the half-of-all-marriages-end-in-divorce figure “appears to be reasonably accurate” (quoted in Luscombe 2010, p. 1).

10 Though some evidence shows that the *distribution* of problems does shift with age. That is, younger children are more likely to suffer *certain* bad effects (e.g., blaming themselves for the divorce), and older children more likely to suffer certain *other* bad effects (e.g., acting out aggressively) on average. But no age group is spared difficulty, and research has not identified a “better” age (all things considered) to go through parents' divorce (Amato 1993).

11 Perhaps they never did line up (see Coontz 2005), but we will focus the bulk of our attention in this article on the difficulties of the present day.

12 When we presented these arguments at a recent seminar, one audience member commented, “We didn’t need evolutionary theory to tell us *that*!”

13 Along with extramarital sex, which tops the list, the other most frequently cited causes of divorce by the couples actually involved are: communication difficulties, general incompatibility, not spending enough time at home, and disagreements over money (Amato and Rogers 1997). We note that adultery considered *on its own*—given the contribution of these other causes—explains (in absolute terms) a minority of breakups, but that clearly many of these causes are interrelated, some more proximate, some more distal.

14 Setting aside communities that already practice “open marriages” or otherwise agree, in advance, that sex outside of the primary relationship is acceptable to both parties. We see nothing wrong, prima facie, with extramarital sex in these sorts of arrangements—rare as they are (see Hunt 1974). Evidence shows, however, that open marriages, where they exist, are typically initiated by just one spouse—the husband—and are only reluctantly agreed to, often under threat of abandonment, by the other spouse—the wife (Buss 1994). So in practice, nonmonogamy may be more ethically problematic than it is in theory.

15 An anonymous reviewer suggested that love drugs could be used to eliminate *jealousy* rather than the impulse to stray (as we go on to propose). For individual couples, this may indeed be a worthwhile strategy, if, within the constellation of their values, it is jealousy that is causing the most trouble by conflicting with their higher-order goals (such as sexual openness, if that is at the top). We cannot set the moral priorities for any given relationship. But in making a more general argument, we note that most couples *as a matter of fact* value sexual fidelity and make an explicit promise to hold to it. Further, this promise may be justified from the perspective of child welfare (as we stated above) since extramarital sex can lead to the formation of extramarital bonds that could drive resources away from existing offspring.

16 It also means we live much longer than is needed for raising children. So, the evolved pair-bonding instincts upon which modern marriages are built have a tendency to dilute long before “death do us part.” Modally, divorces occur at 4 years—the same window of time ancestral babies needed biparental care to ensure survival—with another spike at 7 years—the window needed for a pair of babies (see Fisher 2000).

17 By “we,” we do not mean to include officials in the Catholic Church hierarchy, although we *do* include the large majority of Catholic laypeople who, along with nearly every other demographic in modern Western society, take birth control and reproductive choice to be moral goods (see Greenhouse 2011).

18 This doesn’t mean, of course, that love was the *actual* reason the respondents themselves got married.

19 There is one final marriage value that we have not yet considered in detail—that marriages should be lifelong. We will not take a blanket stand, in this paper, on whether couples should promise to stay together from their wedding day until one or both of them dies. In some cases, divorce is clearly necessary—when abuse is involved, as we stated in Section 1—and in other cases, it may be simply ethically unproblematic: if each member of the married pair is convinced that they would be happier apart, and if there are no children involved, nor any other entangling considerations, then there is no reason to suggest that they *must* stay together against both of their wills. More ethically controversial is the case in which a husband leaves his wife for a younger bride, against the wife's wishes, but out of her control, due to no-fault divorce laws such as are typical in the USA (see Spaht 1998). This case is suspect partly because women's sexual viability declines, on average, much more rapidly than men's—leading to reduced prospects for remarriage on the part of the wife (Buss 1995). Since this asymmetry is due to biological factors, it may violate basic principles of fairness for the husband to exploit it for his own self-interest, after having made a promise of sexual fidelity for better or worse and until death do us part. But we will leave such cases alone. It is neither marriage values nor divorce *in general* that are our concern here, but rather divorce specifically when children are in the picture. This is because the weight of evidence, as we stated earlier, suggests that marriage breakdown, and especially outright divorce, is harmful to children. We think that couples with children, then, may have a special obligation to maintain their relationships in as happy and as healthy a state as possible, if not for life, then at least until the children mature.

20 For example, some couples might be so distant that touching or a physical relationship is no longer possible. Controlled, directed use of the hormone oxytocin (see Section 7) might promote beneficial physical contact in concert with such activities as watching a film together or going for a walk. No biological intervention can manufacture love out of thin air: the administration of any drug would have to be combined with appropriate activities. Under these pharmacologically supported conditions, relationship-promoting behavior might be made easier or more likely to occur—a first step toward restoring a functional and rewarding relationship.

21 The use of MDMA in psychotherapy presents a useful window into the sort of drug administration we envision: short-term use, under controlled conditions, to facilitate states of mind conducive to making progress in relationship health.
